# Musculoskeletal Disorders among Cosmetologists

**DOI:** 10.3390/ijerph6122967

**Published:** 2009-11-27

**Authors:** Alexandra Tsigonia, Dimitra Tanagra, Athena Linos, Georgios Merekoulias, Evangelos C. Alexopoulos

**Affiliations:** 1 Department of Aesthetics and Cosmetology, Technological Educational Institute of Athens, 12210 Athens, Greece; E-Mail: nikal1@hotmail.com; 2 Department of Hygiene and Epidemiology, School of Medicine, Athens University, 11527 Athens, Greece; E-Mail: alinos@med.uoa.gr; 3 Occupational Health Unit, Department of Public Health, School of Medicine, Patras University, 26500 Rio Patras, Greece; E-Mails: dimtanagra@gmail.com (D.T.); gmerek@upatras.gr (G.M.)

**Keywords:** cosmetologists, beauty therapists, aestheticians, beauty salons, musculoskeletal complaints, occupational health, epidemiology, Greece

## Abstract

A cross-sectional study was performed to investigate the relationships between physical, psychosocial, and individual characteristics and different endpoints of low back, neck, shoulder, hand/wrist and knee musculoskeletal complaints among cosmetologists in Athens, Greece. The study population consisted of 95 female and seven male beauty therapists (response rate 90%) with a mean age and duration of employment of 38 and 16 years, respectively. Neck pain was the most prevalent musculoskeletal complaint, reported by 58% of the subjects, while hand/wrist and low back complaints resulted more frequently in self-reported consequences (chronicity, care seeking and absenteeism). Significant relationships were found between self-reported physical risk factors like prolonged sitting, use of vibrating tools, reaching far and awkward body postures and the occurrence of musculoskeletal disorders at various body sites. Among psychosocial variables co-worker support and skill discretion seem to be the most important reflecting organizational problems and cognitive-behavioral aspects. The study results also suggest that effective intervention strategies most likely have to take into account both ergonomic improvements and organizational aspects.

## 1. Introduction

Cosmetologists, aestheticians, beauticians, massage and beauty therapists are synonymous terms referring to people who work in the beauty industry. Their common tasks include facial cleansing, skin, nails and body hydrotherapy and care, anti-wrinkle, pigmentation and acne treatment, make up, depilation, body and face massage, reflexology, aromatherapy, face and body hair removal, *etc.* They usually work in sections of stores selling cosmetics, beauty clinics, centres and salons. They used various chemicals and equipment like steam equipment, depilatory needles, lamps, magnifiers, medicinal and decorative cosmetics. Occupational skin and respiratory disorders, cancer, and disputable reproductive effects have been described among beauty workers, although most studies have focused on hairdressers [1–9]. Paradoxically, very few studies have been identified in a recent search of the international literature on work-related musculoskeletal disorders (MSDs) in the cosmetic industry, but none focus in cosmetologists (beauty therapists) [10–13], although job tasks include the well-established risk factors predisposing towards MSDs [14,15].

A high prevalence of work-related musculoskeletal disorders has been recorded among workers who are exposed to manual labor, work in unusual and restricted postures, repetitive and static work, vibrations, and poor psychological and social conditions [15–20]. In most studies only a few of these risk factors have been taken into account simultaneously. This makes it difficult to appreciate the impact of specific risk factors since most studies did not control appropriately for concurrent risk factors. Since the prevalence and the consequences (e.g., absences and health care use) of MSDs are high, research on the influence of work-related risk factors on the occurrence of MSDs should include its role on aggravation of MSDs, e.g. chronicity, and the consequences [20–24].

The aim of this study was to monitor prevalence and consequences (sickness absence and health care use) and to investigate associations between personal characteristics, physical load, psychosocial factors and general health status with complaints of back, shoulder, neck, hand/wrist and knee among cosmetologists.

## 2. Study Design and Data Collection

It is estimated that more than 750 certified cosmetologists are occupied in the greater Athens area. Employees of more than 50 randomly selected beauty salons in Athens were asked to participate in the study by giving their informed consent. At least one year of work experience in the current position was the only criterion for eligibility for the study. Finally 114 cosmetologists were reached and all agreed initially to participate in the study.

A self-administered questionnaire was distributed by the researchers between April and July 2008. The questionnaire involved information on the respondent’s job and employment history, individual characteristics, physical and psychosocial risk factors at work, general health status, and the occurrence of musculoskeletal complaints. The questionnaire has been tested previously for comprehensibility and relevance and has been used in various settings [20–23].

Individual characteristics and work history included questions on age, anthropometry, sex, family situation, level of education, duration of employment, and previous jobs held. Questions on physical workload concerned manual materials handling, such as lifting and carrying loads, prolonged sitting and standing, awkward working postures in which the back is bent or twisted, repetitive movements and strenuous arm or hand positions. A four-point scale was used with ratings “seldom or never”, “now and then”, “often”, and “always” during a regular workday. The answers “often” and “always” were classified as high exposure. The study subjects also rated their perceived exertion on a Borg-scale ranging from 6 (very light) till 20 (very heavy), with a score of 16 or higher regarded as high perceived exertion [25].

Psychosocial aspects at work distinguished three principal areas: demands, control, and support [26]. *Job demands* were measured by 11 questions on the psychological demands dimension from the Demand/Control model from Karasek *et al.* [27]. The questions were scored on a four-point scale, yielding a sum score for job demands. High demands were related to items such as working fast and hard, excessive work, insufficient time to complete a duty, or conflicting demands. *Lack of control* (decision latitude) was measured by 17 questions of the Decision Latitude dimension from the Demand/Control model [27]. Six items referred to skill discretion, and 11 items to decision authority. The questions were related to creativity, skills, task variety, learning new things, and amount of repetitive work. *Lack of co-worker support* and *Lack of supervisor support* was measured by 18 questions [27]. It included questions about to what degree employees support each other or have conflicts with each other. This was combined with questions about to what extent management is supportive and friendly to the employees. All psychosocial factors were expressed as percentage of the highest possible score, with 0% indicating the best possible situation and 100% the worst possible situation. In the statistical analysis, scores above the median value considered as the presence of psychosocial risk.

The health status of each subject was ascertained with three different outcomes, *i.e.*, perceived general health, need for recovery, and musculoskeletal complaints*. Perceived general health* status was ascertained by 13 dichotomized questions about subjective health complaints, such as respiratory complaints, stomach complaints, regular headache, and tiredness. A sum score was calculated to represent the worker’s actual health situation. This scale had a good internal scale reliability (Cronbach’s α = 0.86) and test-retest reliability (Pearson’s r = 0.76) [28]. *Need for recovery* was measured with 11 dichotomized questions to assess short-term health effects that reflect the worker’s need for recovery at the end of a regular workday. These questions addressed items such as tiredness after work, fatigue, lack of concentration, putting interest in other people, the ability to recover from work, and the influence on work performance [28]. For both general health endpoints subjects with a score above the median value were considered to have a high need for recovery or a moderate/bad general health.

Musculoskeletal complaints were measured using the standardized Nordic questionnaire [29]. Four endpoints for each body site were defined: (i) musculoskeletal complaint of back, neck, or shoulder was defined as pain in the past 12 months, which had continued for at least a few hours during the past 12 months, (ii) chronic musculoskeletal pain in the past 12 months, referred to a complaint that was present almost every day in the preceding 12 months with a minimal presence for at least three months, and (iii) musculoskeletal complaint which led to visit a doctor in the past 12 months, and (iv) musculoskeletal complaint which led to a period of sickness absence in the past 12 months.

### 2.1. Statistical Analysis

Logistic regression analysis was performed to evaluate the influence of individual characteristics, physical and psychosocial risk factors at work, and health status on the occurrence of musculoskeletal complaints. Prevalence odds ratios (PORs) with 95% confidence intervals (95% CI) were calculated as measure of association, adjusted for age and sex. For the initial selection of potential risk factors for musculoskeletal complaints univariate logistic regression analysis was used with a significance level of p < 0.15. Subsequently, all independent variables that showed significant associations were included in the multivariate logistic regression model. Age was always included in each model regardless of its significance. These analyses were carried out separately for all musculoskeletal complaints and their three definitions. Due to the small number of participants borderline significant results (p < 0.06) were also retained in the final models and are also presented. Data analyses were conducted by means of the SPSS for Windows 16.1.0 statistical package.

## 3. Results and Discussion

### 3.1. Response

A total of 114 certified beauty therapists in the city of Athens who had at least one year of experience agreed initially to participate in the study. Finally 106 questionnaires were returned of which four were excluded due to incomplete data (response 89.5%). The principal reasons for non-return were changing shifts and lack of time to complete the forms. Missing values did not exceed 3.1% in any variable, except the item concerning supervisor support.

### 3.2. Baseline Characteristics

Table 1 shows the basic characteristics of the study population (n = 102). The subjects consisted predominantly of women (93.1%), with ages ranging from 21 to 62 years. Less than 50% of the study population had higher (*i.e*., more than two years) specific education on cosmetology. Body mass index was within normal limits for the majority, while 15.7% were overweight (BMI 25–30 kg/m^2^) and only one person had a level of BMI > 30 kg/m^2^. 30% lived alone and 25% were responsible for infants or invalid persons.

The study population consisted mainly of experienced beauty therapists with a mean duration of employment of 16 years (Table 1). The average weekly work time was around 40 hours, however more than 50% worked 45–50 hours, and 9% more than 55 hours a week. Transportation (commuting) time ranged from 5 to 90 minutes, with an average 1 hour per day. Most aestheticians work both first and second shift, which indicate pressure and possible conflicts between work-family lives.

### 3.3. Physical Load, Psychosocial Load, and Perceived Health

The presence of self-reported risk factors for musculoskeletal complaints is reported in Table 2. Manual handling of materials encompass regularly applying force with hands or arms (56%) and operating vibrating hand tools (30%). Repetitive movements of your arms or hands (many times per hour) were reported by more than 80% and awkward back posture, primarily flexion of the back, was reported by approximately 50%. Standing for long periods was reported as a risk factor by 77% of cosmetologists while prolonged sitting by 58%. High perceived exertion was reported by 23% of the study group, while only 6% considers exertion to be low. As far as it concerns the psychosocial risk factors cosmetologists reported heavy load (high demands and low control deriving from low levels of decision authority and skill discretion). Level of co-worker support was medium while support from supervisor was higher although there were only 67 valid responses in this variable.

The self-reported general health showed a high need for recovery (mean score of 53; 100 the worst possible) and a bad/moderate perceived general health status (mean score of 42).

### 3.4. Occurrence of Musculoskeletal Complaints

Table 3 presents the 12-month prevalence of musculoskeletal complaints of back, neck, shoulder, hand/wrist and knee among the 102 cosmetologists. Neck pain was the most prevalent musculoskeletal complaint, reported by 58% of the subjects while chronic neck pain was experienced by 10% workers. Hand and low-back complaints were almost as prevalent as neck pain, but prevalence’s of chronic pain were higher for these body sites. Furthermore hand/wrist and low back complaints resulted more frequently in the consequences under study (visit doctor and absenteeism). Neck and knee pain resulted less in a spell of sickness absence than the other complaints. It seems that regarding the burden of the specific complaints, hand/wrist and back pain followed by shoulder were the most important MSDs among cosmetologists. It is worth mentioning that among those with low-back and shoulder/neck complaints 15–30% undergone physical therapy and 25–70% used medication mostly for shoulder complaints.

### 3.5. Associations between Risk Factors and Musculoskeletal Complaints

In univariate analyses most self-reported physical risk factors (especially the regularly applying force with hands or arms; sitting or standing for long periods and operating vibrating hand tools) were significantly related to the occurrence of low back, shoulder, neck, hand and knee pain (both acute and chronic complaints) as well as with care seeking and sickness absence due to musculoskeletal complaints. Chronic complaints were associated with most physical risk factors with the exception of chronic neck pain, which seems to be connected mainly to vibrating tools. Perceived exertion was found a significant risk factor for low-back, shoulder and knee pain (acute and chronic).

In addition psychosocial aspects were also associated with the occurrence of musculoskeletal complaints. Low skill discretion and decision authority were significantly related with chronic complaints but their relation were inconsistent with care seeking and absenteeism. Work demands exhibited significant relations with shoulder, knee and hand/wrist complaints. Low co-worker support and perceived general health exhibited significant relations with most outcomes under study.

Duration of employment was found significant for chronic shoulder and knee pain. Smoking did not exhibit significant relations while responsibility for a child was found significant for chronic low back pain. Age was found significant for knee and chronic shoulder pain and body mass index was also significant for knee pain.

As far as care seeking due to shoulder and hand/wrist pain, this was mainly affected by physical risk factors, with handling vibrating tools appearing to have a significant relationship to care seeking because of neck pain.

Absenteeism due to musculoskeletal complaints seems to be affected by physical risk factors. Increased body mass index and applying force with hands/arms were related to knee pain absenteeism. Prolonged and strenuous postures were associated with absenteeism from almost any site. Finally perceived exertion was found a significant risk factor for low-back, shoulder and knee pain related absenteeism.

The results of the multivariate analyses on risk factors for the occurrence of complaints, after adjustment for age are shown in Table 4. For each musculoskeletal complaint at least one physical risk factor was important, except for low back pain. They exhibited especially high levels of ORs for hand/wrist complaints. Low co-worker support was significant for low back and hand/wrist complaints while other psychosocial factors did not exhibit any significant relation except in the case of hand/wrist pain. Both job demands and skill discretion were important factors related with hand/wrist complaints. Need for recovery was significant for shoulder, hand/wrist and knee complaints while a perceived bad to moderate general health was significantly associated with low back and neck pain (Table 4).

The multivariate analysis on risk factors for care seeking is shown in Table 5. Care seeking related to knee complaints could not be modeled due to the very small number of participants seeking care due to this complaints (n = 3). Age was important for shoulder and hand/wrist associated care seeking with the older cosmetologists to seek care more frequently (data not shown). High exposure to physical factors was related with increased care seeking due to neck and especially to hand/wrist complaints. Decision authority exhibited significant relations with care seeking due to low back, neck and hand/wrist pain. Co-worker support was related to care seeking due to low-back and shoulder complaints (OR ranged between 3.48 and 3.56). Bad/moderate perceived health was also related to increased care seeking due to shoulder and neck complaints (ORs 6.09–7.64).

The results of the multivariate analyses on risk factors for absenteeism due to musculoskeletal complaints are shown in Table 6. Physical factors were important for absenteeism due to knee (OR 17; p < 0.05) and hand/wrist pain while living alone was associated with low back pain absenteeism. Low co-worker support was found important for low back pain and hand/wrist pain absenteeism. Bad perceived health and a high need for recovery were associated with absenteeism due to low back, neck and shoulder pain.

## 4. Conclusions

In this cross-sectional study involving 102 cosmetologists, we found high prevalence for neck, low back and hand/wrist complaints. Regarding chronicity, care seeking and absenteeism hand/wrist and low back complaints followed by shoulder pain were the most important. Our findings are comparable with those in other sectors like hospitals, industry, *etc.* [20–22]. Our study demonstrates a high prevalence for hand/wrist complaints, mainly as a consequence to high exposure to physical risk factors (work at prolonged sitting and strenuous body postures), something that has already been documented [30–33]. Significant relations were found between self-reported physical risk factors like prolonged sitting, use of vibrating tools, reaching far and awkward body postures and the occurrence of musculoskeletal disorders in various body sites. Considering the nature of cosmetologists’ work, it has to be emphasized that within factors strong interrelations are anticipated. For example “prolonged sitting” may be a proxy for repetitive work that involves wrist/hand flexion and extension. Likewise, “manual handling of vibrating tools” may indicate that they need to work with their trunk severely flexed, which may lead to non-neutral neck posture burdening the neck muscles and vertebrae. This underlines the need for a thorough ergonomic analysis of the activities in cosmetology practice that may lead to musculoskeletal disorders.

Our study took into account concurrent risk factors which were strongly interrelated. In the univariate analyses factors of physical load, psychosocial load, and general health were all associated with musculoskeletal complaints. Due to the small size of the study population, the logistic regression analysis may fail to separate the specific contributions of two risk factors that are strongly correlated. Thus, it should be noted that within the domains of physical load, psychosocial load, and general health, it was to some extent arbitrary which factors were retrieved in the multivariate logistic regression model [34].

Among psychosocial variables co-worker support and skill discretion are proven to be the most important. Co-worker support may express organizational problems and intense work, stressful shifts reflecting the Greek status in the field of small and medium beauty salons. Low co-worker support in this environment is more likely to enhance the physical load on the beauty workers leading to the observed increased prevalence of hand/wrist and low back complaints and the relevant absenteeism and care seeking. These findings are supported by other studies as well [35,36]. Low skill discretion however was related to a smaller possibility for care seeking. It is possible that low skill discretion may be seen as an indication of a proxy measure effect. Highly skilled cosmetologists have more control over the work they do but may have to work extensively with non-neutral postures burdening the neck muscles and vertebrae, which is not necessarily an indication of psychosomatic mechanism of their pain. In addition may be they exhibited a trend to remain in duty (higher satisfaction/feedback) by seeking more for care.

Perceived health and need for recovery were important for most outcomes under study which is well described in many studies [20–23]. A consistent impact in all outcomes under study was held by need for recovery and perceived general health. This finding may reflect the probability that subjects with a moderate general health are more likely to experience musculoskeletal complaints or are more inclined to report musculoskeletal symptoms is troublesome. Alternatively, it may also reflect a subject’s ability to cope when symptoms occurred. Episodes of musculoskeletal pain may affect the perceived general health.

The cross-sectional design of this study does not permit causal inference of the observed associations. The observed associations may have been biased due to different selection processes but the mean duration of employment in the current job of more than 15 years suggest that the study was conducted in a reasonably stable population. Hence, it is expected that selection bias will not have influenced the observed associations to a great extent. However, prospective and larger studies are needed to corroborate the observed associations and the differences in risk factors for occurrence of musculoskeletal complaints and risk factors for aggravation and disability of these complaints. Also personal psychological traits and other possible prognostic factors have not included in this study.

The observed results provide valuable evidence and a basis for further research and policy making among cosmetologists. The study results also suggest that effective intervention strategies most likely have to take into account both ergonomic improvements and organizational aspects.

## Figures and Tables

**Table 1.** Personal characteristics and working experience among cosmetologists (n = 102; 93% women).

	**Mean (SD)**	**%**
Age (y)	38.42 (10.74)	
Body mass index (kg/m^2^)	23.09 (2.86)	
Smokers		46
Higher educational level (%)		47
Family situation (%) Alone Relatives/friends		31 69
Children / invalid persons (%)		25
Duration of employment (y)	15.77 (10.55)	
Working time (hours per week)	43.61 (8.28)	
Transit time (min)	29.19 (15.77)	
Supervision at work		45
Schedule (shifts) (%) Morning (only) Evening (only) Full schedule Irregular schedule		13 4 67 16

**Table 2.** Presence of self-reported risk factors for musculoskeletal disorders.

	**Score**		
	**%**	**Mean**	**SD**
*Physical load*			
Manual handling of materials Strenuous shoulder movements Awkward back posture Prolonged sitting and/or standing High perceived exertion	43 20 64 63 23		
*Psychosocial load*			
Decision authority Skill discretion Work demands Co-worker support Supervisor support		43.01 45.50 53.04 33.28 22.74	26.35 19.96 13.25 19.00 18.83
*General health*			
Need for recovery Perceived general health		53.03 42.12	27.06 20.98

**Table 3.** Prevalence of musculoskeletal complaints in the past 12 months among cosmetologists (n = 102).

	**Low back %**	**Shoulders %**	**Neck %**	**Hand / Wrist %**	**Knee %**
Occurrence in the past 12 months Chronic complaints (>3 months) Visit doctor Complaints with sickness absence	53 19 26 30	35 14 22 17	58 10 18 10	53 18 26 25	28 12 3 11

**Table 4.** Multivariate analysis of self-reported risk factors and prevalence of musculoskeletal complaints in the past 12 months among cosmetologists (n = 102).

	**Low back pain**		**Shoulder pain**		**Neck pain**		**Hand/wrist pain**		**Knee pain**	
	**OR**	**95% CI**	**OR**	**95% CI**	**OR**	**95% CI**	**OR**	**95% CI**	**OR**	**95% CI**
Manual material handling		NS		NS	12.60	2.10 to 75.53			6.37	1.90 to 21.37
Prolonged sitting		NS		NS		NS	55.71	8.75 to 354.93		NS
Strenuous shoulder movements		NS	5.99	1.67 to 21.51		NS	25.34	2.80 to 229.09		NS
High perceived exertion		NS		NS		NS		NS	5.33	1.36 to 20.98
Low skill discretion		NS		NS		NS	0.11	0.03 to 0.48		NS
High job demands		NS		NS		NS	7.62	1.81 to 32.08		NS
Low co-worker support	4.33	1.59 to 11.78		NS		NS	5.06	1.20 to 21.42		NS
High need for recovery		NS	6.77	2.26 to 20.25		NS	4.49	0.96 to 20.90	3.87	1.11 to 13.54
Bad/moderate perceived health	6.46	2.39 to 11.47		NS	9.02	1.63 to 49.94		NS		NS
*Nagelkerke R square*		*0,357*		*0,303*		*0,392*		*0,603*		*0,477*

NS: non significant (p > 0.06)

**Table 5.** Multivariate analysis of self-reported risk factors and seeking physician care due to musculoskeletal complaints in the past 12 months among cosmetologists (n = 102).

	**Low back pain**		**Shoulder pain**		**Neck pain**		**Hand/wrist pain**	
	**OR**	**95% CI**	**OR**	**95% CI**	**OR**	**95% CI**	**OR**	**95% CI**
Manual handling of vibrating material		NS		NS	5.67	1.40 to 22.94		NS
Prolonged body position		NS		NS		NS	6.14	1.50 to 25.17
Awkward body postures		NS		NS		NS	3.84	0.98 to 15,00
Low skill discretion	0.19	0.06 to 0.62		NS	0.13	0.02 to 0.67	0.17	0.04 to 0.66
Low co-worker support	3.56	1.26 to 10.09	3.48	0.95 to 12.80		NS		NS
Bad/moderate perceived health		NS	7.64	1.54 to 37.87	6.09	1.39 to 26.78		NS
*Nagelkerke R square*		*0,206*		*0,402*		*0,346*		*0,465*

NS: non significant (p > 0.06)

**Table 6.** Multivariate analysis of self-reported risk factors and absenteeism due to musculoskeletal complaints in the past 12 months among cosmetologists (n = 102).

	**Low back pain**		**Shoulder pain**		**Neck pain**		**Hand/wrist pain**	
	**OR**	**95% CI**	**OR**	**95% CI**	**OR**	**95% CI**	**OR**	**95% CI**
Living alone vs. living with others	3.22	0.98 to 10.57		NS		NS		NS
Manual handling of vibrating tools		NS		NS	15.25	1.52 to 152.8		NS
Prolonged sitting		NS		NS		NS	25.92	4.33 to 155.23
Low skill discretion		NS		NS	0.09	0.01 to 1.07		NS
High job demands		NS		NS		NS	8.28	2.20 to 31.12
Low co-worker support	8.17	2.53 to 26.34		NS		NS	3.19	0.90 to 11.30
High need for recovery		NS	6.43	1.64 to 25.24	7.26	1.07 to 49.36		NS
Bad/moderate perceived health	3.65	1.13 to 11.75		NS		NS		NS
*Nagelkerke R square*		*0.428*		*0.345*		*0.450*		*0.482*

NS: non significant (p > 0.06)
